# Age‐dependent transcriptional and epigenomic responses to light exposure in the honey bee brain

**DOI:** 10.1002/2211-5463.12084

**Published:** 2016-06-13

**Authors:** Nils Becker, Robert Kucharski, Wolfgang Rössler, Ryszard Maleszka

**Affiliations:** ^1^ Behavioral Physiology and SociobiologyBiozentrumUniversity of WürzburgGermany; ^2^ Research School of BiologyThe Australian National UniversityActonAustralia

**Keywords:** DNA methylation, insect brain, light‐induced gene expression, microRNA, neuronal plasticity

## Abstract

Light is a powerful environmental stimulus of special importance in social honey bees that undergo a behavioral transition from in‐hive to outdoor foraging duties. Our previous work has shown that light exposure induces structural neuronal plasticity in the mushroom bodies (MBs), a brain center implicated in processing inputs from sensory modalities. Here, we extended these analyses to the molecular level to unravel light‐induced transcriptomic and epigenomic changes in the honey bee brain. We have compared gene expression in brain compartments of 1‐ and 7‐day‐old light‐exposed honey bees with age‐matched dark‐kept individuals. We have found a number of differentially expressed genes (DEGs), both novel and conserved, including several genes with reported roles in neuronal plasticity. Most of the DEGs show age‐related changes in the amplitude of light‐induced expression and are likely to be both developmentally and environmentally regulated. Some of the DEGs are either known to be methylated or are implicated in epigenetic processes suggesting that responses to light exposure are at least partly regulated at the epigenome level. Consistent with this idea light alters the DNA methylation pattern of *bgm*, one of the DEGs affected by light exposure, and the expression of microRNA *miR‐932*. This confirms the usefulness of our approach to identify candidate genes for neuronal plasticity and provides evidence for the role of epigenetic processes in driving the molecular responses to visual stimulation.

**Abbreviations**
CBr: central brain
DEG: differentially expressed gene
MB: mushroom body
MG: microglomeruli
miRNA: microRNA
OL: optic lobe

Physiological and behavioral adaptations of an animal in response to novel experiences or to a changing environment are crucial for its fitness 1. One mechanism reflecting adaptation is neuronal plasticity, which is achieved via a complex interplay of environmental stimuli, intracellular signal transduction pathways and molecular mechanisms including DNA methylation, histone modifications and microRNAs (miRNAs) 2, 3, 4, 5. The interplay of these factors and their importance for adaptive behavior remains poorly understood.

Visual stimulation is one environmental factor that has been shown to induce neuronal plasticity in species as diverse as mammals and insects 6, 7, 8, 9. One extensively studied example in this context comes from ocular dominance columns in the visual cortex of mammals, which respond preferentially to input from either one eye or the other. Monocular deprivation during a critical period shifts ocular dominance indicating the plasticity of this system upon environmental changes [reviewd in 9]. But even simple light exposure was shown to result in structural changes in the brain of amphibia 10 and insects 6, 7, including the honey bee 11, 12. A number of studies have associated a few plasticity‐related molecular processes and proteins with visually induced neuronal plasticity, for example, transcription of the immediate early genes *Arc* and *c‐Fos* 13, 14, recruitment of the cAMP pathway including PKA and CREB activity 15, 16, Nogo receptor 1 17, and Rho GTPases 10. However, the precise molecular mechanisms of light‐induced neuronal plasticity and the interplay between different molecular pathways are still unclear.

The European honey bee, *Apis mellifera*, is a valuable model system to investigate this topic due to a sophisticated nervous system, rich behavioral repertoire and pronounced behavioral plasticity. With its sequenced genome and emerging epigenetic tools, the honey bee is becoming an organism of choice in studies aiming at unraveling the molecular mechanisms of environmentally induced neuronal changes underlying behavioral plasticity 18.

Honey bee workers perform age‐related tasks in the colony throughout their adult life 19. Young bees progress through a series of duties within the dark hive until after about 3 weeks of age they begin with foraging activity outside the hive, which they commit to for their remaining life 20. A most important point during adult behavioral maturation is the switch from in‐hive activities to outdoor foraging. This nurse‐to‐forager transition is associated with novel experiences in a rapidly changing environment. As foragers leave the dark pheromone‐filled hive and begin to search for food sources they become more visually guided, particularly for localization of food sources and orientation using visual landmarks and sky‐compass based navigation 21. Therefore, foragers need to optimally adjust their visual system and behavior to novel environments and tasks and thus, adaptive changes in the nervous system of foragers have been described on the neuro‐structural and molecular level.

The transition from nursing to foraging correlates with a volumetric increase in the MB 22, a prominent neuropil in the insect brain involved in sensory integration, memory formation, and spatial orientation 23, 24. The volume expansion depends on age and experience and is mainly caused by the outgrowth of dendrites of the MB intrinsic neurons (Kenyon cells) 25, 26, 27. At the same time, a density decrease (pruning) of synaptic complexes, so called microglomeruli (MG), takes place 27, 28. Most interestingly, exposing adult worker bees to light is sufficient to trigger MG pruning 12. At the molecular level, high‐throughput analyses of the nurse‐to‐forager transition have uncovered transcriptional changes of several hundred genes, some of which are known to modulate synaptic strength and synapse formation 29, 30, 31, 32, 33. This transition to foraging has also been associated with epigenetic changes at the level of DNA methylation and miRNA expression 34, 35. Altogether, these findings illustrate the high degree of neurostructural‐ and molecular plasticity of the honey bee brain upon environmental changes which are partly driven by simple light exposure.

In this study, we have used the honey bee model to investigate environmentally induced brain plasticity at the level of transcription, DNA methylation and microRNA expression. In a broader context, our aim is to understand how sensory stimuli contribute to the genome‐environment interplay that generates strikingly different phenotypes and behaviors without conventional genetic changes.

## Materials and methods

**Figure 1:**
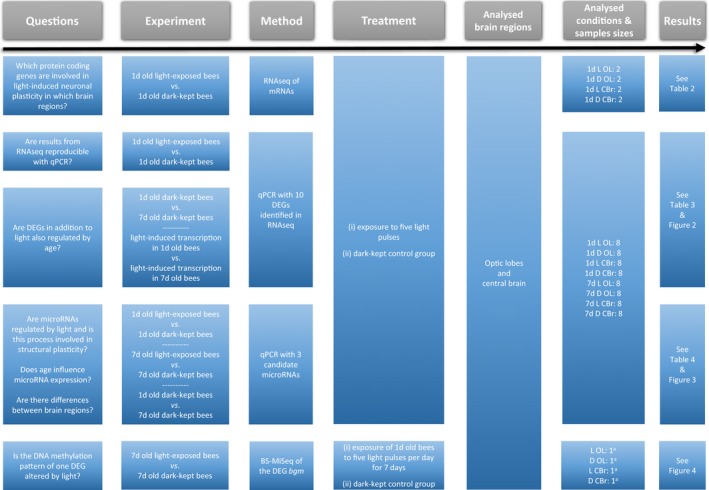
Experimental design. 1d, 1‐day‐old bees; 7d, 7‐day‐old bees; L, light‐exposed bees; D, dark‐kept bees; OL, optic lobe; CBr, central brain. ^a^Four replicates of the light experiment were performed. Eight brain structures (OLs or CBrs) were pooled per sample, whereby two structures derived from each of the four replicates (2 × 4 = 8).

An overview of our aims and experimental designs is shown in Fig. 1.

### Whole transcriptome sequencing (RNAseq)

#### Animals

For RNAseq, newly emerged worker honey bees (*Apis mellifera ligustica*) were obtained from the Australian National University (ANU) apiary in Canberra. Two independent replicates of the following experiment were performed, one in April and one in May 2013. A comb with late pupae was taken from a hive, cleared of any bees, transferred to an incubator and kept at 34.5 °C in complete darkness. To collect age‐matched bees, newly emerging individuals were harvested within a 2 h time window under dim red light conditions. These young bees were transferred immediately as groups of 15 individuals to two wooden cages containing a small tube filled with honey from the same apiary. The caged bees were kept overnight in darkness at 32 ± 1 °C, 30–50% humidity.

#### Light exposure paradigm and sampling point

The next day, one cage of 1‐day‐old bees (~ 24 h, referred as 1d) was exposed to five 45 min lasting pulses of artificial day light [light source: combined fluorescent tubes Repti‐Glo 2.0 15W 45 cm and Repti‐Glo 10.0 15W 45 cm from EXO‐TERRA (Holm, Germany) at 35 cm distance]. Each light pulse was followed by a 75 min dark pause. This light protocol originates from a study with desert ants which aimed at simulating first exposure to light during first orientation (learning) walks 8. In this species, the protocol was shown to induce structural brain plasticity and with the same light program, structural changes were also quantifiable in the honey bee brain after 3 days 8, 12. Our intention in this study was not to mimic light exposure as it occurs during first orientation flights of the honey bee, but solely to use this protocol as a tool to induce structural neuronal plasticity. The control cage remained in darkness. Directly after the fifth and last light pulse bees of the light and the dark group were immediately snap‐frozen in liquid nitrogen and stored until further use at −80 °C. Bees in all experiments were sampled at the same time of day. We choose a sampling point on the first day of light exposure because we assumed that at this time point, a couple of hours after the initial light pulse, molecular processes mediating structural plasticity like transcription would be ongoing.

#### Library preparation

Frozen bees were partly thawed and brains quickly dissected in 50 mm NaCl, 25 mm Tris, 5 mm EDTA, pH 8 (0.5× NTE buffer) as per our standard protocol (see a detailed video recording at https://db.tt/wSj9BBxL). The brains were split into optic lobes (OLs) and the rest referred to as central brain (CBr) and then transferred to separate 1.5 mL Eppendorf tubes kept on dry ice. Five CBrs or five pairs of OLs were pooled per sample. Samples were homogenized for 5–10 s with a plastic pestle (Z359947; Sigma‐Aldrich, Munich, Germany) attached to a hand‐held motorized device. Total RNA was extracted using Trizol and then processed on magnetic beads (Dynabeads; Invitrogen, Waltham, MA, USA) as per recommended protocol with the exception of the number of washes before final elution of mRNA that was increased to five. About 100 ng of rRNA‐depleted mRNA was used for library construction with the NEBNext Ultra Directional RNA Library Prep Kit (#E7420S; NEB, Ipswich, MA, USA) and sequenced on the Illumina MiSeq machine (500 cycles kit MS‐102‐2023; Illumina, San Diego, CA, USA). Transcript variants level estimation‐ RNAseq reads from the GenBank SRA database were queried with 120 bp‐long sequences covering symmetrically all predicted exon 4 3′splice junctions using stand‐alone blast+. Specific junctions were identified and scored by analyzing the resulting alignments; a score was incremented if there was a continuous (ungapped) alignment of minimum 70 nucleotides. Transcript content is estimated as a percentage of a specific junction in all junctions analyzed. *Apis mellifera* genome assembly v.4.5 was used (www.beebase.org). RNAseq data are available at http://dna.anu.edu.au. Libraries were prepared for each treatment group (light, dark) and brain region (OL, CBr) from two independent biological replicates of the experiment, resulting in a sample size of 2 for each condition (light OL, light CBr, dark OL, dark CBr).

### Quantitative real‐time PCR

#### Animals

For quantitative real‐time PCR (qPCR), worker honey bees (*Apis mellifera* var. *carnica*) were obtained from colonies of the apiary at the Biocenter, University of Würzburg, Germany from July to October 2013, and in August 2014. Bee collection and bee handling were performed as described above for the RNAseq experiments with the exception of feeding which was with 50% Apiinvert (Südzucker, Mannheim, Germany), and the time window for collecting newly emerged bees, which was extended to 8 h.

#### Light program and sampling point

The light protocol for 1‐ and 7‐day‐old bees was the same as for RNAseq. For qPCR experiments with 7‐day (7d) old bees, the newly emerged bees were kept for 6 days at 32 ± 1 °C, 30–50% humidity in cages in total darkness before starting the light treatment on the seventh day after eclosion. Sampling again took place directly after the fifth light pulse for both age groups.

#### Sample preparation

Primers (Table 1) for qPCR experiments were designed on the basis of the *A. mellifera* Genome Assembly 4.5. Their specificity could be validated by a blast search against the *A. mellifera* genome, by gel electrophoretic analysis of the PCR products and by a melt curve analysis. Their efficiency (E) was determined in a standard curve analysis by the eppendorf mastercycler ep *realplex* software version 2.2.0.84 (Eppendorf, Hamburg, Germany) with a nondiluted and diluted (1 : 2, 1 : 4, 1 : 8) samples (Table 1). The forward primers for the miRNAs were designed on the basis of the sequences available at mirBase (http://www.mirbase.org/). The forward primer for the noncoding reference RNA *RNU6‐2* (*GB50324*) and reverse primers for miRNA quantification were obtained from the miScript II RT Kit (Qiagen, Hilden, Germany). Note that the provided *RNU6‐2* primer assay was designed against the human sequence (Entrez Gene ID: 26826). The integrity of this primer assay for use in *A. mellifera* could be validated by a blast search with the human *RNU6‐2* sequence against the *A. mellifera* genome, by a gel electrophoretic analysis with the PCR product of the primer assay, and melt‐ and standard curve analysis.

**Table 1:** Primer sequences for qPCR and nested PCR

Symbol	Full name	BeeBase gene ID	Forward‐/reverse primer	Primer efficiency
GB41720	*Uncharacterized LOC727121*	GB41720	CGACCAACACCATGCTACCT/ CGTAACATTCGAACGGCGAC	1.91
GB48020	*Uncharacterized LOC552041*	GB48020	ACGAAGCGATACAACTTACGGT/ CGTATTGCTCTATTCAGTGCGTC	1.9
GB55613	*Uncharacterized LOC100576118*	GB55613	CTGAACGCGACAGAAACGAC/ TCTGATTGGTTCAGAGCGTCA	1.98
*Ip3ka*	*Inositol 1,4,5‐triphosphate kinase 1*	GB41220	GCCGGCCAGTGACGTATTAT/ TTCCACTTCTCTGTAATATCTTGGT	1.93
*Jhbp‐1*	*Take‐out‐like carrier protein (juvenile hormone binding protein‐1)*	GB48492	ACCCAATACACATAGACTGGGA/ GCAGGATTGAATTTCACCGCA	2.35
*L(2)efl*	*Protein lethal(2)essential for life*	GB45913	ACCTTGGGGTGAACTTCTGC/ CCCTCGACGACAACACACTT	1.92
*RpL32*	*Ribosomal protein L32*	GB47227	CGTCATATGTTGCCAACTGGT/ TTGAGCACGTTCAACAATGG	2.07
*Tim2*	*Timeout*	GB41002	TGCAAGTGCTAGACATTCCCAT/ GGACGTTTGTTTTTCGGTTTCG	1.99
*Trim71*	*Tripartite motif‐containing protein 71*	GB48462	TCGTATCCAGGTGTTGACGAT/ ACGATGTTGCCGTCAGGATT	1.99
*Uty*	*Histone demethylase UTY*	GB54595	GTCAACGCATCCAGGGGTAA/ GGTGCTTGGCTCAGATGACT	1.97
*miR‐210*	*miR‐210*	MI0001581 (miRBase.org)	TTGTGCGTGTGACAGCGGCTA/ miScript Universal Primer (Qiagen)	2.08
*miR‐932*	*miR‐932*	MI0005754 (miRBase.org)	TCAATTCCGTAGTGCATTGCAG/ miScript Universal Primer (Qiagen)	2.04
*miR let‐7*	*miR let‐7*	MI0005726 (miRBase.org)	TGAGGTAGTAGGTTGTATAGT/ miScript Universal Primer (Qiagen)	2.01
*RNU6‐2*	*Uncharacterized LOC724988*	GB50324	*RNU6‐2* miScript Primer Assay (Qiagen)	2.01
*bgm*	*Very long‐chain‐fatty‐acid–CoA ligase bubblegum*	GB51580	Outer primers: TTTTTTAATAATTTTAGGTAGTTG/ AATAAATACTTACTTCAAATTTAC Nested primers: GCAGAATTC‐TATTTTATGTTATATATAGTTGGT/ CGCAAGCTT‐CTAATATATTCACAATATATACAC	/

Brain dissections were performed as mentioned for RNAseq. The OLs and the CBrs from three brains were pooled, respectively. The sample size for each tested gene is indicated in Table 3. RNA was extracted by homogenizing the tissue with a 5 mm steel bead (Qiagen) in 500 μL Trizol on a Tissue Lyser LT (Qiagen) for 3 min at 40 Hz. Subsequent RNA extraction steps were conducted according to the Trizol manufacturers' guide. The RNA pellet was resuspended in 20 μL RNAase‐free water by heating the sample at 80 °C for 2 min. RNA concentration and purity was measured with a μCuvette G1.0 (Eppendorf) in a BioPhotometer plus (Eppendorf). RNA integrity was determined for a few samples by gel electrophoretic analysis.

cDNA was synthesized from mRNA with the QuantiTect Rev. Transcription Kit (Qiagen) according to the manufacturers' guide. One microgram total RNA was used as the starting material. In the final step, the cDNA was diluted 1 : 10 by adding 180 μL TE‐buffer (10 mm Tris, 1 mm EDTA, pH 8). cDNA for miRNA analysis was synthesized with the miScript II RT Kit (Qiagen) according to the manufacturers' guide. One microgram total RNA as starting material and the miScript HiFlex Buffer were used. The resulting cDNA was diluted 1 : 5 by adding 80 μL TE‐buffer.

For relative quantification of mRNA levels via qPCR, 2 μL of the respective diluted template cDNA was mixed with 10 μL KAPA SYBR FAST qPCR MasterMix (peqlab, Darmstadt, Germany), 200 nm of the forward‐ and reverse primer each, and RNAse‐free water to fill up to a final volume of 20 μL. qPCR was run on an Eppendorf Mastercycler ep gradient s realplex² (Eppendorf) with the following program settings: 5 min at 95 °C, followed by 40 cycles of denaturation at 95 °C for 15 s, annealing at 60 °C for 20 s, and extension at 72 °C for 30 s. Melt curves were accessed with the following program: 95 °C for 15 s, followed by rapid cooling to 60 °C and then heating to 95 °C in increments in 20 min. *RpL32* (GB47227) was used as a reference gene in each qPCR run. Each sample was analyzed in technical triplicates. Ct‐values were determined with the default settings by the cycler's software (eppendorf mastercycler ep *realplex*). For relative quantification of microRNA levels via qPCR, 2 μL of the respective diluted template cDNA was mixed with 10 μL KAPA SYBR FAST qPCR MasterMix, 500 nm of the forward primer, 500 nm of the reverse miScript Universal Primer (Qiagen), and water to fill up to a final volume of 20 μL. The same qPCR program was used as described above, except for the annealing temperature, which was at 55 °C. *RNU6‐2* (GB50324) served as a reference noncoding RNA.

To determine whether two groups show a statistically significant difference in the expression level of a respective gene, first the normalized ct‐values (ct_norm, tar_) of the respective target gene from each sample was calculated by subtracting the ct‐value of the reference gene (ct_ref_) from the ct‐value of the target gene (ct_tar_): ct_norm, tar_ = ct_tar_ − ct_ref_. Second, the normalized ct‐values of the target gene from each replicate of one test group were compared to the normalized ct‐values of the target gene of a second group via an independent *t*‐test with the statistics program ibm ^®^ spss ^®^ statistics 21 (IBM, Armonk, NY, USA).

The relative expression ratios (*R*) and standard errors were calculated with the Pfaffl‐method 36, 37.

Samples were prepared for each treatment group (light, dark) and brain region (OL, CBr) from eight independent biological replicates of the experiment, resulting in a sample size of eight for each condition (light OL, light CBr, dark OL, dark CBr), and gene (see Table 3).

### Bisulfite sequencing with MiSeq

#### Animals

Bees used for bisulfite sequencing with MiSeq (BS‐MiSeq) were obtained from colonies of the apiary at the Biocenter, University of Würzburg in August 2014.

#### Light exposure paradigm and sampling point

Newly emerged bees were transferred to cages and exposed for 7 days, instead of the usual 1 day, to light pulses. After the fifth light pulse of each day, the bees remained in the dark overnight as described in 12. An age‐matched control group was kept in the dark. Bees were sampled after the last pulse of the seventh day. As nothing is known about the dynamics of DNA methylation in the honey bee, we decided to extend the light program to 7 days to ensure enough time for the establishment of quantifiable changes in the DNA methylation pattern.

#### Library preparation

Bisulfite sequencing was performed as previously described 38, 39 with the following adjustments. For each treatment group (light, dark), 8 MBs and 8 pairs of OLs, respectively, were pooled. The brains for this experiment derived from four independent biological replicates of the experiment, whereby two brains from each replicate were included in the pool. DNA from the four pools (light OL, light CBr, dark OL, dark CBr) was extracted with the NucleoSpin^®^ Tissue XS kit from Machery‐Nagel according to the manufacturers' protocol. Two microgram DNA was used for initial bisulfite conversion. Nested PCR was conducted with primers indicated in Table 1, which flank four CpGs in *bgm*. For library preparation 250 ng of amplicons for each tested group were applied to the NEBNext^®^ DNA Library Prep Master Mix for Illumina^®^, and NEBNext^®^ Multiplex Oligos for Illumina^®^ Index Primers Set 1–4 were used for the different samples.

### Prediction of putative target genes of *miR‐932*

Targets of *miR‐932* were bioinformatically predicted as previously described in 40.

### Phototaxis assay

Newly emerged bees from the apiary at the Biocenter, University of Würzburg were collected in September 2015, separated into four groups, and transferred to cages and exposed to the same light protocol as for the molecular studies. The four groups were (a) bees exposed to light pulses on the first day after eclosion (1d light), (b) an age‐matched dark‐kept control group (1d dark), (c) bees kept in a dark incubator for 6 days before exposure to light pulses on the seventh day after eclosion (7d light), and (d) an age‐matched dark‐kept control group (7d dark). Bees were tested for phototaxis on the day after light treatment to provide a close temporal frame to the molecular studies which may allow an interpretation of the potentially altered phototaxis by light‐induced molecular changes.

Phototaxis was tested in an arena described previously 41, 42. In short, the arena is a lightproof circular construction with 28 cm diameter. Green light emitting LEDs of different relative intensities (12.5%, 25%, 50%, 100%) were installed in the walls with two LEDs of the same intensity positioned opposite to each other. Movements of the bee were recorded via an infrared camera. The bees were put in the dark arena and given 2 min to adapt. Then the lowest intensity LED was switched on. Whenever the bee reached the LED, it was turned off and the opposite LED of the same intensity was switched on. This procedure was repeated four times for each intensity. A bee moving between the two LEDs in a directed manner in at least one of the four trials for the respective light intensity was counted as positive phototaxis for that intensity. Significance was calculated with the Chi‐squared test in ibm ^®^ spss ^®^ statistics 21.

## Results

### Light affects the transcription of protein‐coding candidate genes for neuronal plasticity

For a hypothesis‐free approach of finding genes with transcriptional changes affected by light exposure, we performed two independent RNAseq experiments using mRNA extracted from the OLs and the CBr of 1‐day‐old bees exposed to light and kept in darkness. Although a few hundred genes have shown a detectable level of transcriptional change, many differences became very small after combing the two RNAseq datasets and such genes were not counted as differentially expressed. Only genes with around twofold change in the same direction in both RNAseq datasets were considered further to lower the risk of reporting false positive hits. In experiment 2, a few genes show a very high induction (indicated as 100) suggesting that precise timing is one factor affecting the level of light‐inducible transcripts. This approach has identified 52 genes between the two treatment groups (Table 2). The list of DEG contains genes belonging to a few functional categories: (a) neuronal plasticity (*bgm* 43 *, Cnpy‐1* 44, *Ip3ka* 45), (b) epigenetic functions (histone demethylase *Uty* 46, *histones H3 and H4* 47, and *Trim71* 48 *),* (c) metabolism/energy flux (*GB42985 – n‐acetylneuraminate lyase* 49 *, GB45023 – alpha‐tocopherol transfer protein* 50 *, GB55050 – solute carrier family 26 member 6* 51), and (d) signal transduction (*GB55043 – glutamate receptor, ionotropic kainate 2* 52). A relatively large proportion of DEGs (nine of 52 [17%]) falls into the fifth unknown/novel category. Twelve of 52 DEGs have been shown to be methylated and are predicted to be regulated at the epigenome level.

**Table 2:** Light‐induced DEGs in the OLs and CBr identified with RNAseq

Gene ID	*R* (Log2 ratio)		Methylated	General function
	Experiment 1	Experiment 2		
**Optic lobes**				
GB55613^a^	6.10	100.00		
*Uty* (GB54595)^a^	−1.22	−1.29	Yes	Histone H3K27 demethylase
GB45148	1.14	1.77		Vitamin A‐related
GB45147	1.28	3.13	Yes	Vitamin A‐related
GB45024	0.69	1.00		Vitamin A‐related
GB45023	0.57	2.66		Vitamin A‐related
*Ip3ka* (GB41220)^a^	2.30	1.20	Yes	IP3 kinase
GB42985	3.53	1.96		Pyruvate lyase
*Tim2* (GB41002)^a^	2.32	1.47	Yes	Timeless
GB43805	1.20	1.80		Metallo‐endopeptidase
GB46312	2.86	2.37		Cuticular protein
GB55396	1.28	3.16		Unknown
*Cnpy‐1* (GB50831)^a^	2.02	2.45	Yes	Neurite outgrowth enhancer
*Trim71* (GB48462)^a^	1.30	1.42		E3 ubiquitin‐protein ligase
GB43732	1.13	1.80		Serine/threonine‐proteinkinase
GB44871	2.38	2.36		GglycineN‐methyltransferase
GB47279	3.50	3.60		Cytochrome P450
GB43514	3.04	100.00		Lipase, memberH
GB49843	3.39	2.79		Neuronal PAS domain protein
GB54962	1.12	4.19		Unknown
GB42197	3.73	1.09		Unknown
*Histone H3* (GB47484)	1.41	1.68		Histone H3
GB47382	1.31	3.19		HistoneH4
GB41720	1.98	2.74		Pleckstrin
*Jhbp‐1* (GB48492)^a^	1.07	1.32	Yes	Take‐out
GB42467	2.91	7.10		Phototransduction
GB42673	1.54	2.16		RDH10/retinoldehydrogenase
GB43649	1.31	1.17		Chloride channel
GB55043	2.57	1.87		Kainate glutamate receptor
GB43823	2.83	4.72	Yes	Chemosensory protein CSP1
GB41593	3.22	3.28	Yes	Cell migration regulator
GB40046	1.43	100.00		Neuronal mt transport protein
GB55050	100.00	100.00		Transmembrane transporter
GB41277	1.14	3.29	Yes	Light‐induced ubiquitylation
GB45365	1.08	1.88		Transmembrane transporter
GB47948	1.47	3.08		Myosin light chain kinase
GB41720	1.98	2.74		Plekstrin
GB51220	1.20	1.32		Cytochrome b‐561
GB40552	2.69	3.02		Unknown
GB45910	1.23	1.31		Crystallin
GB45906	1.05	1.07		Crystallin2
GB46514/GB46515	1.19	1.46	Yes	Acetylcholinesterase (bothloci)
GB44095	1.60	3.11		Cation channel
GB42227	4.30	3.59		Homeobox‐related
*bgm* (GB51580)	1.91	1.73	Yes	Acyl‐CoA synthetase
GB41339	2.22	100.00		Acid phosphatase
GB52448	2.75	2.53		Unknown
GB53210	2.22	2.57		Unknown
GB47697	1.79	1.04		Unknown
GB41709	2.20	1.21		Unknown
**Central brain**				
GB41720^a^	1.52	1.00		Low density lipoprotein receptor adapter
GB48020^a^	−1.04	−0.76		Flocculation protein FLO11
*L(2)efl* (GB45913)^a^	1.26	1.51		Protein lethal(2)essential for life
GB44549	−1.43	−1.59		Glucose oxidase
GB41310	2.69	1.30		Actin
GB45796	−2.96	−1.26		Major royal jelly protein 3
GB41309	1.92	2.58		Unknown
GB41307	1.90	2.43		Unknown

*R*, relative expression ratio (Log2); 100.00: Because there is virtually no expression in one condition the increase is shown as 100. ^a^Genes checked with qPCR.

Of special interest for our study are genes listed in the first functional category (a) because of their direct implication in neuronal plasticity. For example, *Ip3ka* encodes a protein that accumulates in dendritic spines in the hippocampus after long‐term potentiation in mice and after spatial learning tasks in rats 53, 54. *Ip3ka* knock‐out mice show a decrease in dendritic‐spine density in the dentate gyrus and defects in memory performance 53. Furthermore, it is proposed that *Ip3ka* modulates dendritic structures by its interaction with f‐actin 45. *Cnpy‐1* may also contribute to structural plasticity in the honey bee brain as the overexpression of this gene leads to neurite outgrowth in cell cultures 44. Finally, *bgm* is important for the correct formation of the OLs in adult flies and is suggested to play a role in myelinogenesis 43, 55.

In the OLs, all DEGs except *Uty* show upregulation after light induction suggesting that light exposure tends to activate transcription of most genes in the optic lobes. The role of *Uty* gene in the honey bee is not known, but K27 methyl mark on histone H3‐K27 is part of transcriptional regulation in mammals. Therefore, it is likely that in our experiment, light‐influenced responses of *Uty* also imply similar regulatory function 46.

**Figure 2:**
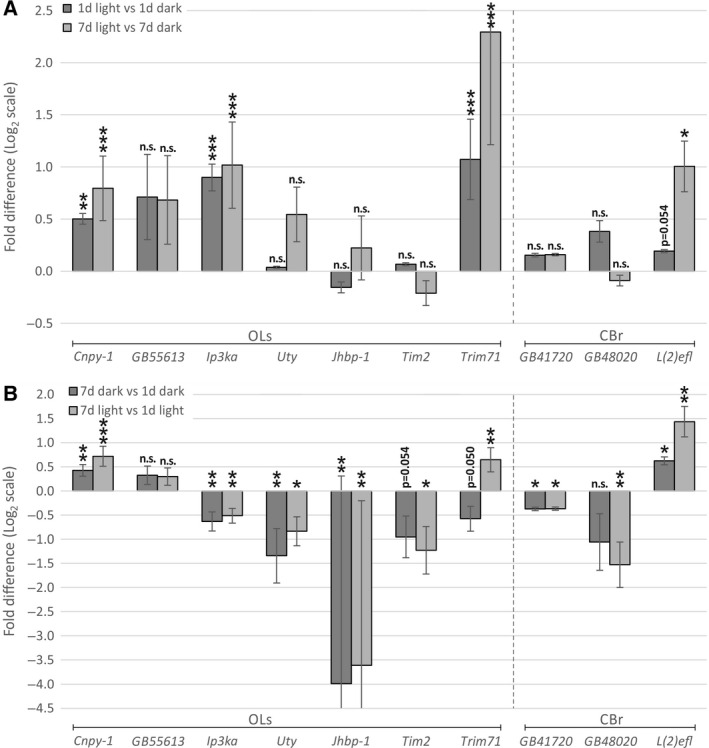
Effect of light and age on gene expression examined by qPCR. (A) 1‐ and 7‐day‐old honey bees were exposed to light pulses for 1 day and light‐dependent gene expression in the OLs as well as in the CBr was compared with an age‐matched dark‐kept control group. (B) Age‐dependent gene expression between 1‐ and 7‐day‐old honey bees was compared in the OLs and CBr for dark‐kept and light‐exposed animals. Ratios were determined by qPCR. 1d, 1‐day‐old bees; 7d, 7‐day‐old bees; OL, optic lobes; CBr, central brain; *P‐value < 0.05; **P‐values < 0.01; ***P‐value < 0.001; n.s.: *P*‐value ≥ 0.05.

We chose seven of the 52 DEGs in the OLs for additional qPCR analyses using material derived from independent replicates of the experiment. Of these seven genes, five (*Cnpy‐1, GB55613, Ip3ka, Tim2,* and *Trim71*) show the same direction of differential expression as found with RNAseq, whereas two genes (*Uty, Jhbp‐1*) show an opposite direction (Table 3 and Fig. 2). Of the consistent five genes, three (*Cnpy‐1, Ip3ka, Trim71*) show a statistically significant differential expression between the two treatment groups. We also have tested these seven genes for differential expression between the treatment groups in the OLs of older bees (7‐days of age) with qPCR. Again, *Cnpy‐1, Ip3ka,* and *Trim71* show a statistically significant difference between the light‐ and the dark group (see Table 3 and Fig. 2) in the qPCR study.

**Table 3:** Effect of light exposure and age on the transcription of protein‐coding genes in the OLs and CBr determined by qPCR

Symbol	Light *vs*. Dark						7‐day‐old *vs*. 1‐day‐old					
	1d light/1d dark			7d light/7d dark			7d dark/1d dark			7d light/1d light		
	*R* (Log2)	*n*	*P*‐value	*R* (Log2)	*n*	*P*‐value	*R* (Log2)	*n*	*P*‐value	*R* (Log2)	*n*	*P*‐value
Optic lobes												
*Cnpy‐1*	0.51	8	**	0.80	8	***	0.42	8	**	0.72	8	***
*GB55613*	0.71	8	n.s.	0.69	8	n.s.	0.32	8	n.s.	0.30	8	n.s.
*Ip3ka*	0.90	8	***	1.02	8	***	−0.62	8	**	−0.51	8	**
*Uty*	0.04	8	n.s.	0.55	8	n.s.	−1.36	8	**	−0.84	8	*
*Jhbp‐1*	−0.15	4	n.s.	0.23	4	n.s.	−4.06	4	**	−3.64	4	**
*Tim2*	0.07	8	n.s.	−0.22	8	n.s.	−0.94	8	0.054	−1.22	8	*
*Trim71*	1.07	8	***	2.29	8	***	−0.58	8	0.050	0.65	8	**
Central brain												
*GB41720*	0.15	8	n.s.	0.16	8	0.130	−0.38	8	*	−0.36	8	*
*GB48020*	0.38	8	n.s.	−0.09	8	n.s.	−1.06	8	n.s.	−1.51	8	**
*L(2)efl*	0.19	8	0.054	1.01	8	*	0.62	8	*	1.43	8	**

*R*: relative expression ratio (Log2); *n*: samples size for each group; 1d: 1‐day‐old bees; 7d: 7‐day‐old bees; *P*‐value: independent *t*‐test comparing normalized ct‐values of the two respective groups; *P‐value < 0.05; **P‐values < 0.01; ***P‐value < 0.001; n.s.: *P*‐value ≥ 0.05.

In contrast to the OLs, in the CBr of 1‐day‐old bees, RNAseq has revealed a much lower number of only eight DEGs (Table 2). From this list, one gene (*L(2)efl*) was reported to have a direct function in neuronal plasticity. *L(2)efl* is linked to Charcot‐Marie‐Tooth neuropathy 56, and known to mediate neurite growth in sensory neurons 57. This may be due to its interaction with the cytoskeleton, especially with f‐actin 58.

From the eight DEGs in the CBr, three (*GB41720*,* GB48020*,* L(2)efl*) were tested with qPCR with material from independent replicates of the experiment. *GB41720* and *L(2)efl* show a tendency toward a higher expression in the light group, which was in line with the results from RNAseq (Table 3 and Fig. 2). The differential expression of *L(2)efl* is close to a significant *P*‐value (independent *t*‐test: *P‐*value = 0.054). *GB48020* shows an opposite direction of expression as seen with RNAseq. Next, we have examined the same three genes for differential expression between the light and the dark group in the CBr of 7d‐old bees via qPCR. *L(2)efl* shows a statistically significant 2.01‐fold higher expression in the light group (Table 3 and Fig. 2). *GB41720* tends to be slightly upregulated (1.12‐fold) in the light group as well, but a significance level was not reached (independent *t*‐test: *P‐*value = 0.130). No significant differential expression is seen for *GB48020*.

Altogether, 70% (seven of 10) of the DEGs identified via RNAseq and tested with qPCR show the same tendency of change in both methods, which confirms the robustness of our assay. Possible reasons for the 30% discrepancy may have resulted from experimental differences between the two methods. Bees for RNAseq derived from Canberra (Australia), belong to the subspecies *ligustica,* and were fed with honey, whereas bees for qPCR came from Würzburg (Germany), are *carnica* and fed with a sugar solution. Therefore, it seems likely that the divergence may be explained by a differential behavioral or physiological state of the two groups of bees.

### Age affects the transcription of protein‐coding candidate genes for neuronal plasticity

Comparison of the candidate gene expression levels in the OLs between the two age groups (1‐ and 7d‐old bees) by qPCR reveals age‐related differences. Four of seven tested candidate genes are expressed significantly different between the 7d dark group and the 1d dark group (*Cnpy‐1, Ip3ka, Uty, Jhbp‐1*), and two genes (*Tim2* and *Trim71*) show a strong tendency toward differential expression (Table 3 and Fig. 2). *Ip3ka, Uty, Jhbp‐1*,* Trim71,* and *Tim2* are expressed at lower levels in 7‐day‐old bees with only *Cnpy‐1* showing higher expression.

In the CBr, age‐related differences in candidate gene expression also are apparent for the three tested genes: *GB41720*,* L(2)efl,* and *GB48020*. Comparing the expression levels of the 1‐ and 7‐day‐old dark‐kept bees reveals a significantly lower expression of *GB41720* and a strong, but nonsignificant, trend toward a lower expression of *GB48020* in the 7‐day‐old group (Table 3 and Fig. 2). In contrast, the expression of *L(2)efl* in 7‐day compared to 1‐day‐old bees is significantly higher.

Most interestingly, age appears to affect the amplitudes of light‐induced gene transcription. The light‐induced expression of *Cnpy‐1, Ip3ka, Trim71,* and *L(2)efl* is more pronounced in 7‐day‐old bees (Table 3 and Fig. 2). This is particularly obvious for *Trim71* with 2.33 times higher levels of light‐induced expression in the OLs of 7‐day compared with 1‐day‐old bees (*R*_7d light/7d dark_/*R*_1d light/1d dark_).

### Light‐, age‐ and brain‐compartment related expression of candidate microRNAs

**Figure 3:**
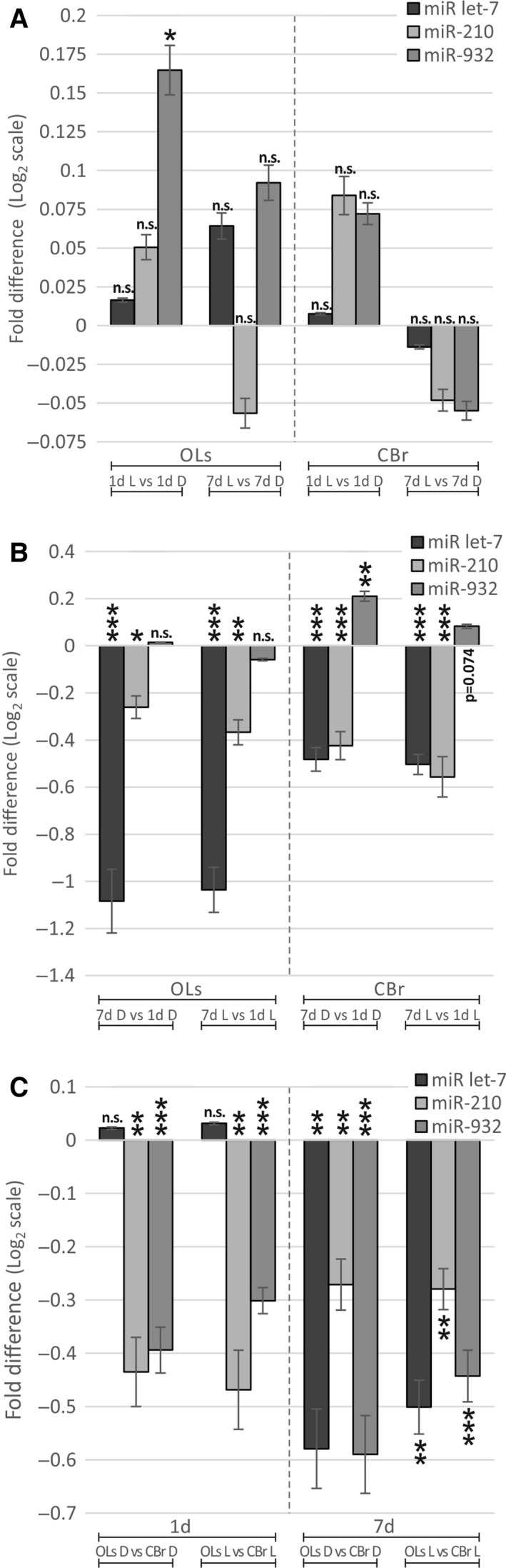
Effect of light, age and brain compartment on miRNA expression examined by qPCR. (A) 1‐ and 7‐day‐old honey bees were exposed to light pulses for 1 day and light‐induced miRNA expression in the OLs as well as in the CBr was compared with an age‐matched dark‐kept control group. (B) Age‐dependent miRNA expression between 1‐ and 7‐day‐old honey bees was compared in the OLs and CBr for dark‐kept and light‐exposed animals. (C) Brain compartment‐dependent miRNA expression was compared between the OLs and the CBr in 1‐ and 7‐day‐old light‐exposed and dark‐kept honey bees. OL, optic lobes; CBr, central brain; 1d, 1‐day‐old bees; 7d, 7‐day‐old bees; D, dark‐kept bees; L, light‐exposed bees; *P‐value < 0.05; **P‐values < 0.01; ***P‐value < 0.001; n.s.: *P*‐value ≥ 0.05.

Since in the OLs, both light exposure and age appear to strongly influence the expression of *Trim71*, a known target of the microRNA *let‐7* (*miR let‐7*) 59, we asked whether the expression levels of this miRNA correlate with *Trim71* levels. Our qPCR analysis has not revealed any light‐inducible effects on *miR let‐7* in both the OLs and CBr of 1‐ and 7‐day‐old light‐exposed and dark‐kept bees (Table 4 and Fig. 3). However, age strongly affects the expression levels. In the OLs of 7‐day‐old bees, the *miR let‐7* level was half as low as in 1‐day‐old bees (0.47 fold), which correlates with more than twice as high (2.33) light‐induced *Trim71* expression in 7‐day compared with 1‐day‐old bees. In other words, low *miR let‐7* levels correlate, in an age‐dependent manner, with relatively high light‐induced *Trim71* levels and vice versa. Interestingly, a similar age‐dependent correlation was described for *C. elegans,* in which age‐dependent expression of *miR let*‐*7* differentially regulates axon growth potential through its interaction with *lin‐41* (the homolog of *Trim71*). High levels of *miR let‐7* in old neurons inhibit *lin‐41* expression leading to a decline in axon plasticity, whereas in young neurons low levels of *miR let‐7* result in unhampered *lin‐41* expression maintaining axon plasticity 48. Therefore, in the honey bee brain age‐dependent *miR let‐7* levels may be a critical factor determining the extent or onset of environmentally induced neuronal plasticity mediated by *Trim71*.

**Table 4:** Effect of light exposure, age, and brain compartment on microRNA expression determined by qPCR

Symbol	Light *vs*. Dark											
	OLs						CBr					
	1d light/1d dark			7d light/7d lark			1d light/1d dark			7d light/7d dark		
	*R* (Log2)	*n*	*P*‐value	*R* (Log2)	*n*	*P*‐value	*R* (Log2)	*n*	*P*‐value	*R* (Log2)	*n*	*P*‐value
miR let‐7	0.01	8	n.s.	0.07	8	n.s.	0.01	8	n.s.	−0.01	8	n.s.
miR‐210	0.06	8	n.s.	−0.06	8	n.s.	0.08	8	n.s.	−0.04	8	n.s.
miR‐932	0.16	8	*	0.10	8	n.s.	0.07	8	n.s.	−0.06	8	n.s.

	7‐day‐old *vs*. 1‐day‐old											
	OLs						CBr					
	7d dark/1d dark			7d light/1d light			7d dark/1d dark			7d light/1d light		
miR let‐7	−1.09	8	***	−1.03	8	***	−0.47	8	***	−0.49	8	***
miR‐210	−0.27	8	*	−0.36	8	**	−0.42	8	***	−0.56	8	***
miR‐932	0.01	8	n.s.	−0.06	8	n.s.	0.21	8	**	0.08	8	0.074

	Optic lobes *vs*. central brain											
	Age: 1d						Age: 7d					
	Dark OL/dark CBr			Light OL/light CBr			Dark OL/dark CBr			Light OL/light CBr		
miR let‐7	0.03	8	n.s.	0.03	8	n.s.	−0.58	8	**	−0.49	8	**
miR‐210	−0.43	8	**	−0.47	8	**	−0.27	8	**	−0.29	8	**
miR‐932	−0.40	8	***	−0.30	8	***	−0.60	8	***	−0.43	8	***

*R*: relative expression ratio (Log2); *n*: samples size for each group; 1d: 1‐day‐old bees; 7d: 7‐day‐old bees; OL: optic lobes; CBr: central brain; *P*‐value: independent *t*‐test comparing normalized ct‐values of the two respective groups; *P‐value < 0.05; **P‐values < 0.01; ***P‐value < 0.001; n.s.: *P*‐value ≥ 0.05.

We have quantified the expression levels of two further miRNAs in the OLs and the CBr of 1‐ and 7‐day‐old light‐exposed and dark‐kept bees, *miR‐923* and *miR‐210*, which have been linked to brain functions in the honey bee 40, 60, 61. The expression of *miR‐932,* but not *miR‐210,* shows a significant light effect (Table 4 and Fig. 3). In the OLs, the expression of *miR‐932* is 1.12‐fold higher in 1‐day‐old light‐exposed bees compared with the age‐matched dark‐kept ones (independent *t*‐test: *P*‐value = 0.036). This light effect persists in the OLs of 7‐day‐old bees, but with no statistical significance (independent *t*‐test: *P*‐value = 0.107). As *miR‐932* shows a transcriptional response to light, we predicted its putative targets (Table 5). These include *GB44947* and *GB45281* which have reported functions in neuronal plasticity in other organisms. *GB44947* homologs (*Doublecortin*) are involved in proper f‐actin formation, microtubule stabilization, and neuronal migration 62. The homolog of *GB45281* (*E3 ubiquitin‐protein ligase Hyperplastic discs*) regulates *hedgehog* and controls photoreceptor differentiation in *Drosophila* and, therefore, is a good candidate for adaptation processes in the honey bee eye in response to sensory stimuli 63. Although no differential expression of *GB44947*0 or *GB45281* has been detected in our study, it is conceivable that *miR‐932* affects their regulations at specific time points after light exposure. Furthermore, miRNAs have the ability to subtly fine‐tune gene transcription at distinct subcellular locations (i.e. at synapses or even dendrites), which would be unlikely to detect with our approach extracting total RNA from entire brain areas 64.

**Table 5:** Putative targets of *miR‐932*

Honey bee			Fly ortholog	
Gene ID	Symbol	General function	Symbol	General function
GB50397	/	Unknown	PDZ‐GEF	PDZ domain‐containing guanine nucleotide exchange factor
GB44947	LOC726454	Similar to CG13467‐PA	DCX‐EMAP	Doublecortin‐domain‐containing echinoderm‐microtubule‐associated protein
GB44221	Noc2	Nucleolar complex protein 2	CG9246	
GB54520	/	Unknown	/	/
GB47477	LOC726348	Similar to peroxisomal biogenesis factor 6	Pex6	Peroxin 6
GB54355	yps	Ypsilon schachtel	yps	Ypsilon schachtel
GB55860	/	Unknown	Ect4	Ectoderm‐expressed 4
GB55364	/	Unknown	Ptp99A	Protein tyrosine phosphatase 99A
GB45281	hyd	E3 ubiquitin‐protein ligase hyd	hyd	Hyperplastic discs
GB41610	/	Unknown	/	/
GB44526	LOC551919	Similar to Paxillin CG31794‐PC, isoform C	Pax	Paxillin

### Light affects DNA methylation of *bgm*

**Figure 4:**
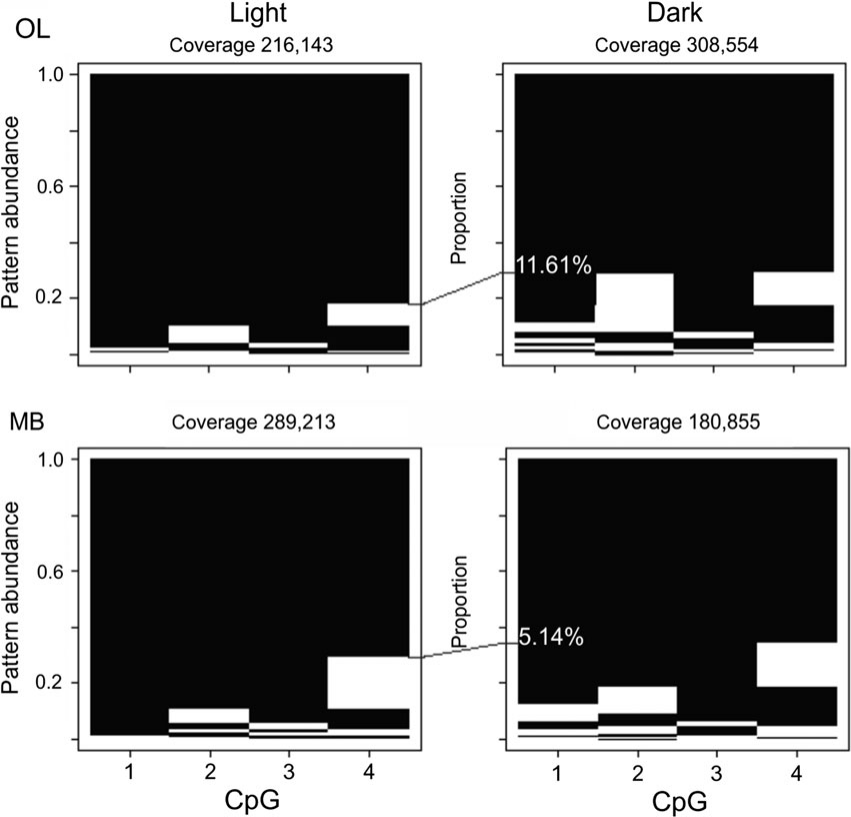
Effect of light on the methylation pattern of *bgm*. Methylation patterns in *bgm* revealed by deep amplicon sequencing. Each row represents a methylation pattern (black: methylated CpGs, white: not methylated CpGs), the height of each pattern is proportional to the pattern's abundance. *bgm* amplicons were amplified from both OLs and MBs using light‐exposed and dark‐kept bees. After normalizing pattern frequencies several distinct and highly abundant methylation patterns have been detected. The pattern proportions are sorted from the most abundant at the top to the least abundant at the bottom. The number of sequenced reads for each situation is shown above each panel. OL, optic lobes; MB, mushroom bodies.

Eleven DEGs from our study are known to be methylated. To examine if DNA methylation changes are associated with light exposure in these DEGs, we have used ultra‐deep bisulfite sequencing of gene‐specific amplicons 38, 39. This method has the capacity to generate up to 1 million reads per amplicon and its resolving power is sufficient to visualize all condition‐specific methylation patterns that may be associated with dozens of distinct cell types, even if methylation levels in certain cell types are very low. We selected one of the DEGs, *bgm*, as the illustrator gene because in previous analyses it has shown a relatively high level of methylation in a short region of DNA spanning 4 CpG sites (see Table 1 for primers flanking this genomic region). The protein encoded by *bgm* plays a central role in brain long‐chain fatty acids metabolism and myelinogenesis, and in correct development of the OLs in adult flies 43, 55. It also has a role in global epigenetic control of transcription because it supplies acetyl‐CoA for histone acetylation by histone acetyltransferases 65. As shown in Fig. 4, *bgm* methylation patterns are responsive to light exposure, especially in the OLs where there is more than 11% more methylation seen at all four CpGs in a certain proportion of patterns, but with CpGs #2 and 4 most affected. The light influence also is detectable in the MBs, but the increase in methylation in this neuropil is less pronounced (5.14%). Given the very high sequencing coverage in each sample, it is likely that patterns showing the highest methylation dynamics represent a few specific cell types that are primarily responsible for processing light signals in both brain compartments.

### Age and possibly light treatment affect phototaxis

**Figure 5:**
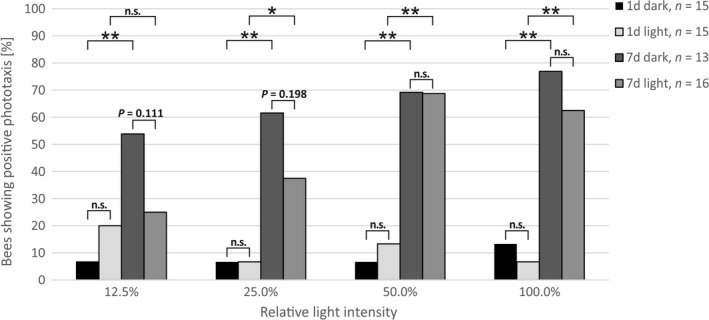
Effect of light and age on phototaxis. 1‐ and 7‐day‐old bees, which have been exposed to light pulses for 1 day, and age‐matched dark‐kept control bees were tested for positive phototaxis at four different relative light intensities. 1d, 1‐day‐old bees; 7d, 7‐day‐old bees; *P‐value < 0.05; **P‐values < 0.01; n.s.: *P*‐value ≥ 0.05.

Given the effect of light exposure on the transcription of several genes in an age‐dependent manner, we were interested whether light treatment and age have an effect on vision‐related behavior, in particular phototaxis. We have found that for each of the four tested light intensities a higher percentage of 7‐day‐old bees responded positively to the light source in comparison to 1‐day‐old bees (see Fig. 5). Prior light treatment does not significantly alter positive phototaxis in 1‐day‐old bees, but a trend for decreased positive phototaxis was found in 7‐day‐old bees. At the lowest intensity (12.5%), more than twice as many (2.1‐fold) 7‐day‐old dark‐kept bees exhibit positive phototaxis compared with 7‐day‐old light‐treated bees. At an intensity of 25%, the difference in positive phototaxis between the two groups decreased to 1.6‐fold, and was finally similar at the two highest intensities. We do not have a conclusive explanation for this phenomenon and can only speculate that somehow prior light treatment either reduces the reception or perception of low light intensities, or reduces the motivation for walking towards low light intensities.

## Discussion

To date, very few studies have examined the effects of direct light exposure on gene regulation in the context of neuronal plasticity with the majority of prior work in this field focusing on various aspects of the circadian rhythm. In one relevant study on light‐inducible transcriptome in zebra fish, 117 light‐regulated genes have been identified of which most (90) were upregulated 66. This is in line with our findings demonstrating an upregulation of 51 genes with only one being downregulated. One possibility for this relatively small number of light‐inducible genes is that transcriptional responses to light are chronological with distinct networks activated at different times. This explanation is partly confirmed by the observed age‐dependent gene activities. Alternatively, light in general, may affect the expression of a relatively small number of genes. Also, it is likely that different light paradigms and sampling points may result in quite distinct sets of DEGs. For example, the lack of immediate early genes in our dataset, previously reported to respond to light exposure 13, 14, can be attributed to our specific experimental conditions. Among DEGs reported in this paper, *Ip3ka, Cnpy‐1, bgm,* or *L(2)efl* participate directly in neuronal plasticity involving neurite outgrowth and synapse morphology 43, 44, 45, 57. Thus, these genes could also be critical in mediating dendritic outgrowth in the honey bee brain upon neuronal activation that occurs during the transition from nursing to foraging or after artificial light exposure 12, 25. This idea is supported by the fact that *L(2)efl* also has been found to be upregulated in the heads of foragers compared with nurses 29. Interestingly, a study on daily transcript oscillation in *Drosophila* has reported that light‐induced transcripts belong to similar broad categories as those identified in our experiments (inositol metabolism, ubiquitin pathway, solute transport) suggesting that in insects, light may induce similar molecular responses 67.

A surprising outcome of our study is the relatively low number of DEGs in the CBr compared with the OLs. The most prominent structural remodeling upon light stimulation occurs in the MBs, manifested by MG pruning 12. We expected this plasticity to be reflected by pronounced transcriptional changes in the CBr, in which the MBs contribute to over 50% of all cells 68, 69. However, as MG elimination in the MBs is due to a pruning of projection neuron boutons which have their cells bodies in the medulla and lobula of the OLs, transcriptional changes reflecting strengthening or weakening of MG may in fact occur in the OLs. Furthermore, the higher number of DEGs in the OLs may indicate severe neuronal plasticity in this region, which so far has not received much attention as it is not as easily quantifiable. However, an electron microscopy study has revealed synaptic plasticity of photoreceptor neurons in the lamina after manipulation of the visual environment 11.

Several of our candidate DEGs are part of the epigenetic machinery controlling gene expression either via DNA or chromatin modifications, i.e. histone demethylase *Uty,* histones *H3 and H4* or *Trim71* 46, 47, 48. Flexible epigenetic mechanisms modulate coordinated gene expression in a context‐dependent manner by acting as the genome‐environment interface. For example, histone modifiers like *Uty* have been shown to affect the expression of a number of plasticity‐related genes 46. Using this mechanism, adult honey bee workers could modulate brain networks to optimize their responses to new environments or to new tasks associated with behavioral maturation, or with light exposure.

We also provide seminal evidence for the role of DNA methylation in regulating light‐inducible neuronal plasticity in the honey bee. In insects, DNA methylation appears to modulate the transcript levels and also participates in alternative splicing 70, 71. Several DEGs identified in this study are known to be methylated, including DEGs with reported plasticity functions like *Cnpy‐1*,* Ip3ka, and bgm*. The connection between visual system and DNA methylation dynamics has been confirmed in this study by showing light‐induced increases in *bgm* methylation levels. Given the reported role of *bgm* in neuronal plasticity, it is likely that the observed methylation changes serve as responsive genomic marks adjusting environmentally driven expression 43, 55. Our findings add to the body of evidence implicating DNA methylation in brain functions in this insect that already includes behavioral transition to foraging 35 and memory formation 72.

Another interesting outcome of our study is a strong age dependence of light‐related differences in the transcription of a number of candidate DEGs. For example, the amplitudes of light‐induced transcription of *Ip3ka, Cnpy‐1, Trim71,* and *L(2)efl* are higher in 7‐day‐old bees compared with 1‐day‐old bees. One possibility is that these age‐dependent differences in transcriptional responses of neuronal plasticity genes to light are important for proper behavioral maturation of adult workers; for example, when they switch to foraging tasks, which is assumed to never happen before they are 4–5 days old (own observations and 73). Younger bees may not be developmentally programmed to participate in foraging and their responses to light exposure are predictably less flexible. Indeed, behavioral consequences of age‐ and environment‐dependent gene expression tested by our phototaxis experiments support this notion. A much higher proportion of 7‐day‐old bees show positive phototaxis compared with 1‐day‐old bees suggesting that bees at different developmental states exhibit distinct behaviors upon light exposure that correlates with differential expression of relevant neuronal genes. Age‐dependent differences in the expression of plasticity‐related genes identified in this study are also apparent when comparing the basal expression levels of the dark‐kept control groups between 1‐ and 7‐day‐old bees. This result speaks for an endogenous mechanism regulating the chronologic expression of light‐responsive neuronal genes during adult maturation. Young bees progress through a series of tasks within the hive which gradually brings them into closer proximity to the hive entrance and light exposure 20. It is likely that this behavior is partly driven by increased phototaxis, and that our observed age‐dependent expression of DEGs serves as a molecular regulation of this behavior.

Our findings also complement recent discoveries implicating miRNAs in brain function. The expression of one miRNA, *miR‐932,* is affected by light. This miRNA was previously shown to have an effect on long‐term memory formation in the honey bee possibly by its direct interaction with the actin gene *Act5c* 61. We have predicted one additional potential target of *miR‐932,* namely *Doublecortin* (*GB44947*) that also is known to interact with f‐actin strengthening the idea that *miR‐932* participates in structural plasticity via its interaction with the cytoskeleton at the level of synapses. These small epigenetic regulators are considered proximate factors mediating age‐dependent differences in the amplitude of light‐induced transcription of neuronal genes. In *C. elegans,* the reciprocal inhibition of *Trim71* and *miR let‐7* depends on age and ultimately determines different degrees of axonal plasticity at different ages 48. Based on the age‐dependent negative correlation between *Trim71 and miR let‐7* levels uncovered in our study, a similar mechanism controlling the onset or degree of neuronal plasticity in an age‐dependent manner seems possible in the honey bee brain. Indeed, it has been suggested that differentially expressed miRNAs, including *miR let‐*7, have a role in developmentally regulated behavioral changes in the honey bee during the transition from nursing to foraging 34. We propose that one role of *miR let‐7* and possibly other miRNAs in this behavioral transition involves the refinement of brain networks in expectation of foraging, or after orientation flights when they collide with the external world. This idea is strengthened by the fact that in the honey bee, miRNAs are predicted to predominantly target neuronal genes 40.

The specific roles of cellular responses to light are certain to be complex, likely warranting years of future research. The findings presented here signify the importance of investigating dynamic regulation of both gene expression and epigenetic modifiers in behavioral changes brought about by the perception of environmental stimuli. The honey bee system allows an unparalleled experimental transition, from transcriptomes and epigenomes to neural circuitry to sophisticated behaviors, all under entirely natural environmental conditions.

## Author contributions

WR, NB and RM conceived the study and designed the experiments. NB performed all lab work. RK analyzed RNAseq and MiSeq data. NB wrote the draft of the manuscript. RM and WR edited the manuscript.
